# Al^18^F-NOTA-FAPI-04 PET/CT for diagnosis of breast cancer and its correlation with pathological features: A single-center retrospective study

**DOI:** 10.1371/journal.pone.0342769

**Published:** 2026-02-12

**Authors:** Yi Wang, Lijuan Feng, Limeng He, Nan Liu, Hao Wang

**Affiliations:** 1 Department of Nuclear Medicine, Zigong Fourth People’s Hospital, Zigong, Sichuan, China; 2 Department of Nuclear Medicine, Sichuan Provincial People’s Hospital, University of Electronic Science and Technology of China, Chengdu, Sichuan, China; Università degli Studi di Brescia: Universita degli Studi di Brescia, ITALY

## Abstract

**Objective:**

To systematically evaluate the diagnostic efficacy of Al¹⁸F-NOTA-FAPI-04 PET/CT in breast cancer and explore the correlation between tracer uptake parameters and pathological features of breast cancer based on single-centre, retrospective data.

**Methods:**

This single-center retrospective study enrolled 58 female patients with suspected primary breast cancer who underwent Al¹⁸F-NOTA-FAPI-04 PET/CT before surgery or core needle biopsy (February 2023–November 2025). Lesion maximum/mean standardized uptake values (SUVmax/SUVmean) were measured via region-of-interest analysis. Semi-quantitative positivity thresholds (SUVmax > 2.5 or tumor-to-background ratio ≥ 1.5) were used for lesion classification. Diagnostic efficacy (sensitivity, specificity, accuracy) was calculated with pathological results as the gold standard, and 95% confidence intervals (CIs) were determined via the Clopper–Pearson exact method. Differences in uptake parameters across pathological types, molecular subtypes, and immunohistochemical (ER/PR/HER2/Ki-67) statuses were analyzed using Mann–Whitney U or Kruskal–Wallis H tests.

**Results:**

Of 58 patients, 49 had breast cancer and 9 had benign lesions. Al¹⁸F-NOTA-FAPI-04 PET/CT achieved a sensitivity of 95.9% (95% CI: 86.3%–99.5%), specificity of 88.9% (95% CI: 51.9%–99.7%), and accuracy of 94.8% (95% CI: 85.8%–98.9%). All 53 primary lesions were detected (detection rate: 100%), and the detection rate for 87 lymph node metastases was 96.5%. SUVmax/SUVmean of malignant lesions (median [IQR]: 13.20 [9.55–17.85]/8.15 [5.68–10.92]) were significantly higher than those of benign lesions (2.13 [1.56–2.89]/1.35 [0.98–1.86], both *P* < 0.001). No statistically significant differences in uptake parameters were observed across pathological types, molecular subtypes, or ER/PR/HER2/Ki-67 statuses (all *P* > 0.05), though numerical trends existed; this may be attributed to Type II error due to small subgroup sizes and insufficient statistical power (29.7%–62.3% for detecting medium-to-large effect sizes). No correlation was found between lesion size and uptake parameters (*r* = 0.186 for SUVmax, *r* = 0.165 for SUVmean, both *P* > 0.05).

**Conclusion:**

Al¹⁸F-NOTA-FAPI-04 PET/CT exhibits high sensitivity and accuracy for breast cancer diagnosis and lymph node metastasis detection, with uptake independent of blood glucose levels. Its core clinical roles include complementary initial diagnosis in high-risk subgroups (dense breasts, diabetes), primary nodal staging, and problem-solving in equivocal cases. However, the lack of significant correlation between uptake parameters and pathological features should be interpreted cautiously due to potential Type II error. These characteristics make it a versatile imaging tool for breast cancer diagnosis and staging in regional tertiary care settings, with further validation needed in larger, balanced cohorts.

## Introduction

Breast cancer is the most frequently diagnosed malignancy among Chinese women; its incidence is rising year by year and the age of onset is steadily decreasing. More than one million women in China are currently living with the disease, and approximately 300,000 new cases are diagnosed annually, constituting a major threat to female health and family quality of life [1,2]. Accurate early detection and precise staging are pivotal for improving prognosis and tailoring therapeutic strategies, and they directly determine long-term survival and quality of life.

Currently available imaging modalities have inherent limitations: breast ultrasonography has low sensitivity for microcalcifications [3]; mammography performs poorly in dense breasts; magnetic resonance imaging offers excellent soft-tissue contrast but is time-consuming, expensive, and associated with a non-negligible false-positive rate [4,5]. ^18^F-FDG PET/CT, the most widely used functional imaging technique, provides whole-body evaluation and plays an important role in initial staging; however, its sensitivity is compromised by tumour differentiation and blood glucose levels, and it frequently misses low-metabolic subtypes such as luminal A breast cancer or invasive lobular carcinoma [6]. Moreover, ¹⁸F-FDG cannot reliably differentiate inflammatory lesions from metastatic lymph nodes, a clinical dilemma that remains unresolved [7].

Fibroblast activation protein (FAP) is highly expressed in >90% of cancer-associated fibroblasts derived from epithelial tumours, whereas expression in normal tissues is minimal, rendering it an ideal target for tumour-specific imaging [8]. FAP-targeted radiotracers are independent of blood glucose, exhibit high tumour uptake, and show low background activity in normal organs. They outperform ¹⁸F-FDG in multiple malignancies, including breast cancer subtypes with suboptimal FDG avidity [9]. Among these tracers, ¹⁸F-labelled FAPI tracers possess a favourable physical half-life (110 min) and are more amenable to large-scale clinical production and distribution than their ⁶⁸Ga-labelled counterparts, offering superior translational value for widespread clinical application [10]. Recent advances in FAP-targeted molecular imaging have further expanded its potential, covering early detection, staging, treatment response monitoring, and even guiding targeted therapeutic interventions, which is expected to significantly advance personalized oncological care for breast cancer [11].

Existing investigations are predominantly multicentre pilot studies with limited sample sizes or comparative analyses among multiple tracers; consequently, robust, single-centre, large-cohort validation data are lacking [12]. For regional tertiary centres such as Sichuan Provincial People’s Hospital, no standardized protocol for Al¹⁸F-NOTA-FAPI-04 PET/CT (hereafter FAPI-PET) in breast cancer imaging has been established. Moreover, published data on the association between tracer uptake parameters and core pathological characteristics—including histological subtype and molecular phenotype—remain inconsistent, highlighting the need for clarification with single-centre pathological cohorts to optimize precision diagnosis and treatment planning [11]. Critically, the specific clinical scenarios where FAPI-PET can provide incremental value—including initial diagnosis, nodal staging, or problem-solving in equivocal cases—have not been clearly defined. This study aims to address this gap by identifying situations where FAPI-PET enhances diagnostic certainty, refines staging, or alters therapeutic decisions. Additionally, region-specific data on the tracer’s biodistribution and optimal diagnostic thresholds in the Sichuan population are lacking—information critical for adapting this advanced imaging technique to local clinical practice.

Leveraging the clinical resources of Sichuan Provincial People’s Hospital, the present single-centre retrospective study was designed to (i) determine the sensitivity, specificity, and overall accuracy of FAPI-PET for breast cancer detection; (ii) characterize its in-vivo biodistribution in the local population; and (iii) explore correlations between quantitative uptake parameters and tumour histology, molecular subtype, and immunohistochemical biomarkers.

## Materials and methods

### Patients

This retrospective study, approved by the Institutional Review Board of Sichuan Provincial People’s Hospital (Approval No. 328, 7 August 2024), enrolled consecutive female patients (≥ 18 years) with suspected primary breast cancer who were initially evaluated at the same institution between February 2023 and November 2025. Given the retrospective design, minimal risk to participants, and use of de-identified data (with no direct identification of individuals), the IRB explicitly waived the requirement for individual informed consent. The retrospective data collection for patients enrolled between February 2023 and August 2024 was fully covered by the aforementioned ethical approval, as the committee explicitly authorized the inclusion of eligible de-identified patient data throughout the entire study period without requiring separate exemption.

**Fig 1:**
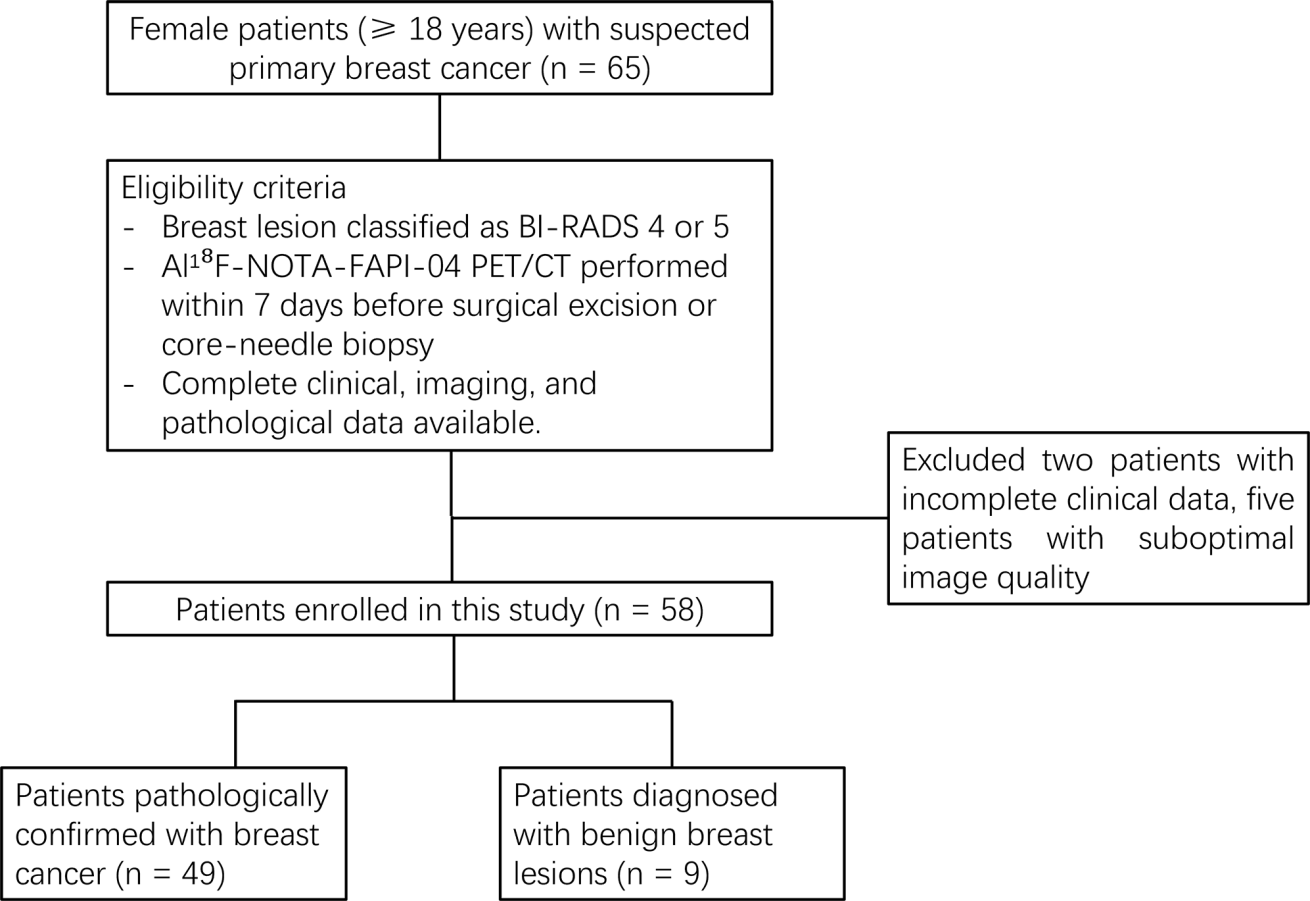
Flow diagram of patient inclusion and exclusion process.

Eligibility criteria were: (i) breast lesion classified as BI-RADS 4 or 5 on ultrasound, mammography, or magnetic resonance imaging with initial suspicion of malignancy; (ii) FAPI-PET performed within 7 days before surgical excision or core-needle biopsy (median interval: 3 days, IQR: 2–5 days); and (iii) complete clinical, imaging, and pathological data available. Patients were excluded if they had (i) incomplete clinical data; (ii) prior breast surgery, radiotherapy, chemotherapy, or targeted therapy; (iii) pregnancy or lactation; or (iv) inability to tolerate PET/CT or suboptimal image quality precluding reliable analysis. The electronic medical-record database was accessed for research purposes on 10 December 2025. Throughout and after data collection, the authors had access only to de-identified information; no data that could directly identify individual participants (e.g., names, national identification numbers, or hospital registration numbers) were available to the research team. To minimize selection and referral bias, all consecutive patients meeting the eligibility criteria were enrolled without discretionary exclusion, ensuring the cohort is representative of suspected primary breast cancer cases referred to our tertiary care institution for definitive imaging and pathological evaluation. The patient inclusion and exclusion process is illustrated in a flow diagram (Fig 1).

### PET/CT imaging protocol

Patient preparation and image acquisition were performed in accordance with standard clinical protocols. Prior to intravenous injection, radiochemical quality control of Al¹⁸F-NOTA-FAPI-04 was conducted in compliance with the European Association of Nuclear Medicine consensus guidelines for radiopharmaceutical production and release. Radiochemical purity was analyzed by high-performance liquid chromatography equipped with UV (254 nm) and radio-detectors, using a C18 column (4.6 × 250 mm, 5 μm) with a mobile phase of acetonitrile:0.1% trifluoroacetic acid (v/v = 40:60) at a flow rate of 1.0 mL/min. Only batches with radiochemical purity ≥ 95% and no detectable free ¹⁸F⁻ (retention time < 2 min) were used for patient administration. After intravenous injection of 3.70 MBq kg ⁻ ¹ Al¹⁸F-NOTA-FAPI-04 adjusted for body weight (median total injected activity: 246.5 MBq, interquartile range [IQR]: 221.8–273.3 MBq), whole-body PET/CT was performed on a Siemens Biograph mCT Flow 64 system at 90 ± 10 min post-injection. CT parameters were: 120 kV tube voltage, automated tube-current modulation, 3 mm slice thickness, 2 mm increment, and 0.8 pitch. PET acquisition used FlowMotion technology with an average bed-speed resulting in a total scan time of ~12 min. Images were reconstructed with TrueX + TOF (2 iterations, 21 subsets), yielding 3 mm slice thickness and 2 mm interval. All datasets were transferred to a Syngo TrueD workstation for multimodal review.

### Image interpretation

Two board-certified nuclear-medicine physicians (each with >5 years of experience) independently interpreted the images in a fully blinded fashion. All disagreements between the two readers were resolved by consensus with a third senior reader who had > 10 years of specialized experience in nuclear medicine imaging. Necrotic and cystic areas within lesions were visually identified and excluded during regions of interest (ROI) delineation to avoid underestimating tracer uptake. For lesions <1 cm in maximum diameter, a fixed-diameter ROI (5 mm) was used for standardized uptake values (SUV) measurement. For lymph nodes (both metastatic and normal), a standard 1-cm spherical ROI was uniformly applied to ensure consistent quantitative analysis. Three-dimensional ROIs were manually delineated on attenuation-corrected PET images to record the maximum (SUVmax) and mean (SUVmean) SUV for the primary tumour and each metastatic focus. To reduce reader subjectivity, we explicitly explored semi-quantitative thresholds during protocol design: (1) SUVmax cutoffs: We tested SUVmax = 2.0, 2.5, and 3.0 based on prior FAPI-PET literature [10,14] and our center’s preliminary data; SUVmax > 2.5 was selected for balancing sensitivity and specificity. (2) Tumor-to-background ratio (TBR): Defined as lesion SUVmax divided by SUVmax of adjacent normal breast tissue (measured in contralateral normal breast or ipsilateral non-lesional area with matched ROI size); TBR thresholds of 1.3, 1.5, and 1.8 were evaluated, with TBR ≥ 1.5 chosen to minimize false positives from physiological uptake. A lesion was classified as positive based on an objective threshold: SUVmax > 2.5 or SUVmax 1.5 times higher than the SUVmax of adjacent normal breast tissue (whichever was stricter). Lesions meeting this criterion were considered malignant unless clearly attributable to physiological distribution or benign pathology (e.g., typical fibroadenoma with characteristic imaging features). To minimize selection bias, consecutive eligible patients were enrolled strictly per predefined criteria without discretionary exclusion, ensuring the cohort was representative of suspected primary breast cancer cases at our institution. Additionally, the fully blinded independent interpretation and consensus-based resolution of discrepancies eliminated potential bias from clinical or pathological prior knowledge, further enhancing the reliability of diagnostic outcomes.

### Pathological and molecular subtyping detection

Surgical specimens or core needle biopsy samples (obtained using 14-gauge core needles), with a median interval of 3 days (interquartile range [IQR]: 2–5 days) between FAPI-PET imaging and histopathological examination. All specimens provided sufficient tissue volume to support definitive pathological diagnosis and molecular subtyping, with no cases of insufficient tissue leading to underestimation of malignancy. Estrogen receptor (ER), progesterone receptor (PR), human epidermal growth factor receptor 2 (HER2) and Ki-67 status were determined by immunohistochemistry (IHC). ER and PR positivity were defined as ≥1% positively stained nuclei. HER2 was scored according to ASCO/CAP guidelines: IHC 3 + was considered positive; 2 + cases were reflex-tested by fluorescence in situ hybridisation (FISH) and regarded as positive only if gene amplification was present, whereas 2 + /FISH-negative tumours were classified as HER2-low and 0/1+ as negative. Ki-67 > 20% was designated high. Molecular subtypes were assigned as Luminal A, Luminal B, HER2-positive or triple-negative (basal-like) [13].

### Statistical analysis

Statistical analyses were performed with SPSS 26.0. Continuous variables were first tested for normality (Shapiro–Wilk). For consistent presentation across all subgroups and to avoid confusion, median (interquartile range) was used for all continuous uptake parameters (SUVmax and SUVmean) since normality tests confirmed non-normal distribution (SUVmax: *P* = 0.012; SUVmean: *P* = 0.008) in the overall cohort. Non-normally distributed data are reported as median (interquartile range) and were analysed with the Mann–Whitney U test for two groups or the Kruskal–Wallis *H* test for multiple groups. Categorical variables are expressed as frequencies (%). Diagnostic performance was quantified by sensitivity, specificity and accuracy. 95% confidence intervals (CIs) for sensitivity, specificity, and accuracy were calculated using the Clopper–Pearson exact method, which is robust for small sample sizes and binary outcome data (e.g., the limited number of benign lesions [n = 9] in our cohort). A two-tailed *P* value < 0.05 was considered statistically significant. Assuming a sensitivity of 0.95 for FAPI-PET vs 0.85 for FDG-PET, α = 0.05 and power = 0.80, the minimum required sample size was 45 patients; the final cohort of 58 patients met this requirement. A post-hoc power analysis for molecular subtype comparisons was performed using G*Power 3.1.9.7. Assuming a medium-to-large effect size (Cohen’s d = 0.8), α = 0.05 (two-tailed), and subgroup sample sizes (Luminal A: n = 5; Luminal B: n = 28; HER2-positive: n = 6; triple-negative: n = 10), power to detect differences in SUVmax/SUVmean among subtypes ranged from 29.7% (small subgroup comparisons) to 62.3% (largest subgroup comparisons), all below the 80% threshold for adequate power. Additionally, 95% confidence intervals for subgroup uptake parameters were wide (e.g., Luminal A SUVmax: 7.89–19.41; HER2-positive SUVmax: 8.92–18.68), indicating poor precision in estimating subtype-specific values.

## Result

### Demographic and clinical characteristics of patients

A total of 58 female patients were enrolled in this study, with an age range of 41–79 years and a median age of 54 years (interquartile range [IQR]: 47–62 years). Regarding menopausal status: 21 patients (36.2%) were premenopausal, and 37 patients (63.8%) were postmenopausal. The median body mass index (BMI) was 23.8 kg/m^2^ (IQR: 21.5–26.3 kg/m^2^), with a range of 18.9–29.7 kg/m^2^. Five patients (8.6%) had a history of type 2 diabetes mellitus, all of whom had well-controlled blood glucose (fasting blood glucose < 7.0 mmol/L) at the time of PET/CT examination. Among them, 49 patients were pathologically confirmed with breast cancer, and 9 patients were diagnosed with benign breast lesions (4 cases of fibroadenoma, 3 cases of breast hyperplasia, and 2 cases of inflammatory nodules) (S1 Table).

Among the 49 breast cancer patients, 45 had single primary lesions and 4 had multifocal primary lesions (2 lesions per patient), resulting in a total of 53 primary breast cancer lesions. The pathological types of these primary lesions included invasive ductal carcinoma (41 cases, 83.7%), invasive papillary carcinoma (5 cases, 10.2%), mucinous carcinoma (2 cases, 4.1%), and ductal carcinoma in situ (1 case, 2.0%). According to the molecular subtype classification based on immunohistochemical results: Luminal A type (5 cases, 10.2%), Luminal B type (28 cases, 57.1%), HER2-positive type (6 cases, 12.2%), and triple-negative type (Basal-like type, 10 cases, 20.4%). Among the breast cancer patients, 16 cases (32.7%) were found to have lymph node metastases, with a total of 87 metastatic lymph nodes detected (including 59 axillary lymph node metastases, 9 internal mammary lymph node metastases, and 19 supraclavicular lymph node metastases). The maximum diameter of primary breast cancer lesions ranged from 0.8 cm to 5.2 cm, with a median diameter of 2.3 cm (IQR: 1.6–3.1 cm).

### Diagnostic efficacy of FAPI-PET for breast cancer

Taking the pathological results as the gold standard, FAPI-PET diagnosed 47 of the 49 breast cancer patients correctly, with 2 false-negative cases (both were Luminal A type breast cancer, accounting for 40% (2/5) of all Luminal A cases in the cohort). These two false-negative lesions had a diameter of <1.0 cm (0.8 cm and 0.9 cm, respectively), were located in dense fibroglandular breast tissue [BI-RADS density category 3], and were not adjacent to high physiologic cardiac uptake. Their SUVmax values were 2.3 and 2.4, respectively—below the objective positivity threshold of 2.5 and not meeting the 1.5 × normal tissue uptake criterion. Among the 9 benign lesion patients, 8 were diagnosed correctly, with 1 false-positive case (inflammatory nodule with SUVmax of 3.2, exceeding the 2.5 threshold and showing 1.8 × uptake relative to adjacent normal tissue; the patient underwent follow-up ultrasound and FAPI-PET/CT 4 weeks after anti-inflammatory therapy, which showed complete resolution of the lesion and normalized tracer uptake).

The sensitivity, specificity, and accuracy of FAPI-PET for diagnosing breast cancer were 95.9% (95% confidence interval [CI]: 86.3%–99.5%), 88.9% (95% CI: 51.9%–99.7%), and 94.8% (95% CI: 85.8%–98.9%), respectively. Notably, the specificity estimate is associated with substantial uncertainty due to the small number of benign cases (n = 9), resulting in an extremely wide 95% CI (51.9%–99.7%). This limits the reliability and generalizability of the reported specificity, and the result should be interpreted with extreme caution to avoid overgeneralization to broader clinical populations. The wide 95% CI for specificity is due to the small sample size of benign lesions, which restricts the precision of this metric. In addition, the sensitivity of FAPI-PET for Luminal A breast cancer was 60% (3/5), which is substantially lower than the overall sensitivity of 95.9%. This subgroup-specific performance discrepancy highlights the need for careful interpretation of FAPI-PET results in suspected Luminal A cases.

**Fig 2:**
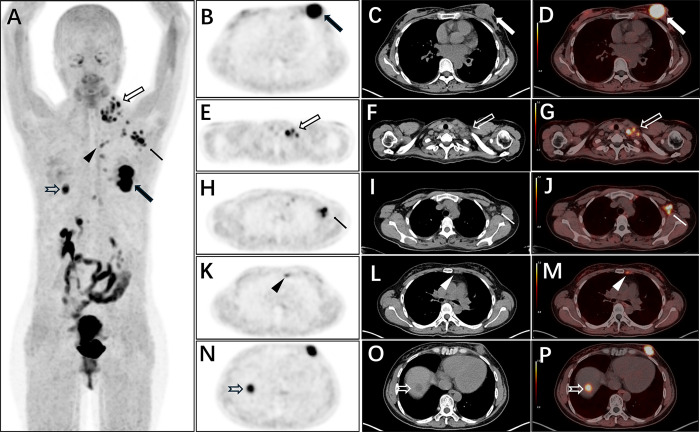
Al¹⁸F-NOTA-FAPI-04 PET/CT images of a 38-year-old female patient with breast invasive ductal carcinoma (molecular subtype: LuminalB). **(A)** Whole-body maximum-intensity projection (MIP) image shows multiple FAPI-avid lesions. Solid thick arrows in axial PET **(B)**, CT **(C)**, and fused PET/CT **(D)** images indicate the primary breast tumor. Open thick arrows in axial PET **(E)**, CT **(F)**, and fused PET/CT **(G)** images denote supraclavicular lymph node metastases. Thin arrows in axial PET **(H)**, CT **(I)**, and fused PET/CT **(J)** images indicate axillary lymph node metastases. Triangles in axial PET **(K)**, CT **(L)**, and fused PET/CT **(M)** images indicate an internal mammary lymph node. Open arrowheads in axial PET **(N)**, CT **(O)**, and fused PET/CT **(P)** images indicate a hepatic metastasis.

**Fig 3:**
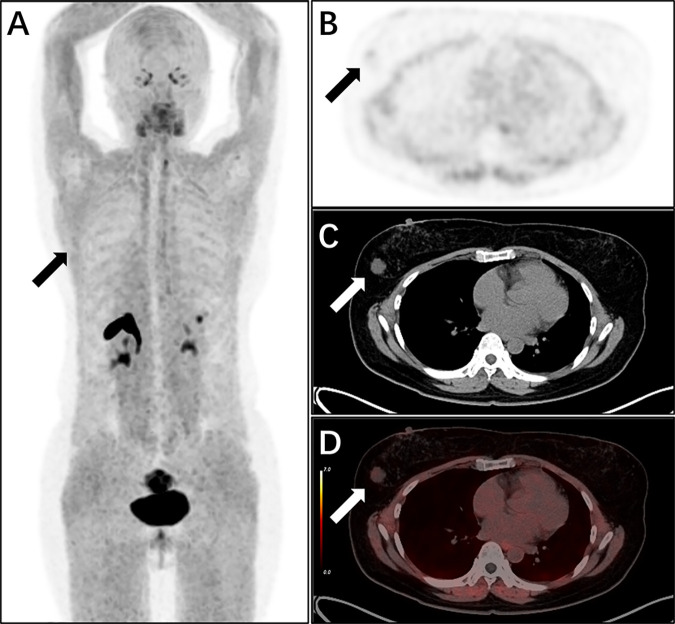
Al¹⁸F-NOTA-FAPI-04 PET/CT images of a 50-year-old female patient with breast mucinous carcinoma (molecular subtype: HER2-positive). **(A)** Whole-body maximum-intensity projection (MIP) image reveals a mildly FAPI-avid lesion (low to moderate tracer uptake) in the right breast. Solid thick arrows in axial PET **(B)**, CT **(C)**, and fused PET/CT **(D)** images localize the primary tumor (mucinous carcinoma, a subtype with indolent biology), which exhibits low tracer uptake (consistent with the subtype’s relatively low cancer-associated fibroblast infiltration).

In terms of lesion detection: all 53 primary lesions of breast cancer (from 49 patients) were accurately identified, with a detection rate of 100%; among the 87 lymph node metastases, 84 were detected, and 3 tiny metastatic lesions (diameter <3 mm) were not identified, with a lymph node metastasis detection rate of 96.5%. The diagnostic efficacy of FAPI-PET for breast cancer is shown in Table 1. Representative images are provided in Figs 2 and 3.

**Table 1:** Diagnostic efficacy of FAPI-PET for breast cancer.

Index	Value	95% CI
**Sensitivity**	95.9% (47/49)	86.3%–99.5%
**Specificity**	88.9% (8/9)	51.9%–99.7%
**Accuracy**	94.8% (55/58)	85.8%–98.9%
**Positive predictive value**	97.9% (47/48)	89.5%–99.9%
**Negative predictive value**	80.0% (8/10)	44.4%–97.5%

### Uptake parameters of lesions and correlation with pathological features

The median (IQR) SUVmax and SUVmean of primary breast cancer lesions were 13.20 (9.55–17.85) (range: 4.82–25.36) and 8.15 (5.68–10.92) (range: 3.25–16.78), respectively, which were significantly higher than those of benign lesions [SUVmax: 2.13 (IQR: 1.56–2.89), range: 1.25–3.52; SUVmean: 1.35 (IQR: 0.98–1.86), range: 0.82–2.15; both *P* < 0.001]. For lymph node metastases, the SUVmax was 9.01 (IQR: 4.92–15.53) (range: 3.65–18.72) and SUVmean was 5.43 (IQR: 3.11–9.65) (range: 2.18–11.36), which were lower than primary breast cancer lesions but significantly higher than the physiological uptake of normal lymph nodes (median [IQR] SUVmax: 1.20 (1.05–1.42), *P* < 0.001). Notably, no statistically significant differences in SUVmax or SUVmean were observed among different pathological types, molecular subtypes, or ER/PR/HER2/Ki-67 expression statuses (all *P* > 0.05) (Tables 2–4). However, numerical trends were observed: (1) Pathological types: Invasive papillary carcinoma had the highest median SUVmax (14.20) vs. invasive ductal carcinoma (13.15) and mucinous carcinoma (12.85); (2) Molecular subtypes: HER2-positive subtype showed a slightly higher median SUVmax (13.80) than other subtypes; (3) Immunohistochemical features: ER-positive tumors had ~ 18% higher median SUVmax (13.15) than ER-negative tumors (11.12), and Ki-67 low-expression tumors (≤20%) had ~ 21% higher median SUVmax (15.60) than high-expression tumors (>20%, 12.85). These numerical differences represent small-to-moderate effect sizes but lacked statistical significance due to limited sample size and power. Additionally, this finding should be interpreted cautiously due to the extremely small sample size of rare subgroups: mucinous carcinoma (n = 2), DCIS (n = 1), and Luminal A molecular subtype (n = 5). These small subgroup sizes, combined with post-hoc power analysis showing insufficient statistical power (29.7%–62.3% for detecting medium-to-large effect sizes), increase the risk of Type II error (false negative), meaning subtle but clinically relevant correlations may exist but were not detected in this cohort.

**Table 2:** Comparison of uptake parameters of primary lesions among different pathological types of breast cancer.

Pathological Type	Number of Cases	SUVmax(Median [IQR])	SUVmean(Median [IQR])
**Invasive ductal carcinoma**	41	13.15 (9.48–17.92)	8.08 (5.62–11.05)
**Invasive papillary carcinoma**	5	14.20 (10.15–18.55)	8.75 (6.20–10.80)
**Mucinous carcinoma**	2	12.85 (9.90–15.80)	7.95 (5.85–10.05)
**Ductal carcinoma in situ**	1	13.20 (-)	8.10 (-)
***H* value**	–	1.826	1.753
***P* value**	–	0.635	0.652

Normality test results (Shapiro–Wilk): SUVmax (*P* = 0.012), SUVmean (*P* = 0.008) — both parameters were non-normally distributed across the overall cohort, so median (IQR) is used for consistent presentation. No significant differences were observed (all *P* > 0.05). The number of cases of invasive papillary carcinoma (n = 5), mucinous carcinoma (n = 2), and ductal carcinoma in situ (n = 1) is ≤ 5. Thus, the statistical analyses for these pathological types are exploratory and require confirmation in larger cohorts.

**Table 3:** Comparison of uptake parameters of primary lesions among breast cancer patients with different immunohistochemical features.

Pathological Feature	Number of Cases	SUVmax(Median [IQR])	*P* Value	SUVmean(Median [IQR])
**ER**	–	–	0.350	–
**-Negative**	16	11.12 (7.80-16.40)	–	7.65 (4.82–10.55)
**-Positive**	33	13.15 (10.60-17.45)	–	8.25 (6.10–11.20)
**PR**	–	–	0.520	–
**-Negative**	20	14.40 (9.85–18.75)	–	8.65 (5.92–11.50)
**-Positive**	29	13.00 (9.55–17.20)	–	8.10 (5.58–10.85)
**HER2**	–	–	0.240	–
**-Negative**	12	10.85 (10.30-12.70)	–	7.05 (6.60-8.35)
**-Low expression**	27	14.00 (8.15-20.10)	–	8.25 (4.62-12.55)
**-Positive**	10	13.50 (12.40-20.90)	–	8.22 (7.12-12.95)
**Ki-67**	–	–	0.270	–
**-Low expression (≤20%)**	13	15.60 (11.25–19.85)	–	9.35 (7.20–11.80)
**-High expression (>20%)**	36	12.85 (9.20–17.15)	–	7.95 (5.38–10.65)

Normality test results (Shapiro–Wilk): SUVmax (*P* = 0.012), SUVmean (*P* = 0.008) — both parameters were non-normally distributed across the overall cohort, so median (IQR) is used for consistent presentation. All exact *P* values are reported, and no statistically significant differences were observed (all *P* > 0.05).

**Table 4:** Comparison of uptake parameters of primary lesions among different molecular subtypes of breast cancer.

Molecular Subtype	Number of Cases	SUVmax(Median [IQR])	SUVmean(Median [IQR])
**Luminal A type**	5	13.65 (9.80–18.25)	8.30 (6.15–10.55)
**Luminal B type**	28	13.20 (9.65–17.75)	8.18 (5.72–10.92)
**HER2-positive type**	6	13.80 (10.55–19.20)	8.40 (6.35–11.25)
**Triple-negative type**	10	13.50 (9.42–17.98)	8.25 (5.50–10.70)
***H* value**	–	1.952	1.886
***P* value**	–	0.587	0.603

Normality test results (Shapiro–Wilk): SUVmax (*P* = 0.012), SUVmean (*P* = 0.008) — both parameters were non-normally distributed across the overall cohort, so median (IQR) is used for consistent presentation. No significant differences were observed (all *P* > 0.05). Note that the number of cases corresponding to pathological types such as invasive papillary carcinoma, mucinous carcinoma, and ductal carcinoma in situ (not listed as independent molecular subtypes here but included in the overall cohort) is ≤ 5. Therefore, the statistical results for rare pathological types/molecular subtype-related analyses are exploratory and need to be validated in larger sample size studies.

### Correlation between uptake parameters and primary lesion size

The correlation analysis between the maximum diameter of primary breast cancer lesions and FAPI-PET uptake parameters showed that there was no significant correlation between the maximum diameter of lesions and SUVmax (*r* = 0.186, *P* = 0.203) or SUVmean (*r* = 0.165, *P* = 0.257).

## Discussion

In this single-center retrospective study, we systematically evaluated the diagnostic performance of FAPI-PET in patients with biopsy-proven breast cancer and explored the relationship between tracer uptake and core pathological features. The results demonstrate that FAPI-PET achieves high sensitivity and overall accuracy for primary tumor detection. Notably, tracer accumulation was independent of conventional histopathological characteristics, underscoring its robustness as a non-invasive imaging biomarker. These findings support the integration of FAPI-PET into the diagnostic workflow of breast cancer in regional tertiary-care institutions.

### Diagnostic efficacy of FAPI-PET

Our data demonstrated that FAPI-PET achieved a sensitivity of 95.9%, specificity of 88.9%, and accuracy of 94.8% for breast cancer diagnosis, which is consistent with recent multicenter studies on FAP-targeted imaging [11]. Notably, all 53 primary lesions were successfully detected by FAPI-PET, and the detection rate for lymph node metastases reached 96.5%, only missing 3 tiny metastases (<3 mm). This superior performance can be attributed to the high and specific expression of FAP in cancer-associated fibroblasts (CAFs) within the tumor microenvironment. It is reported in >90% of CAFs across pan-cancer studies [14] and further validated in breast cancer using ¹⁸F-labelled FAPI tracers [15,16]. This ensures robust tracer accumulation in malignant lesions [14].

FAPI-PET exhibits notable characteristics that enhance its clinical utility: it is independent of blood glucose levels, eliminating the need for strict pre-examination fasting or glucose control and significantly improving clinical convenience, especially for diabetic patients (8.6% of our cohort) who are at risk of breast cancer [10]. Even in patients with type 2 diabetes mellitus, FAPI-PET maintained high diagnostic accuracy without being affected by blood glucose fluctuations, which further supports its clinical applicability in diverse patient populations.

The two false-negative Luminal A lesions were located in dense fibroglandular breast tissue (BI-RADS density category 3) without adjacency to cardiac uptake. Dense fibroglandular tissue may cause partial masking of tracer signals, and combined with the small lesion size (<1.0 cm) and relatively low CAF infiltration in Luminal A subtype (characterized by indolent biological behavior), this may explain the false-negative results.

The high positive predictive value (97.9%) observed in our study indicates that positive by FAPI uptake is highly suggestive of malignancy, which can help clinicians prioritize invasive diagnostic procedures (e.g., core needle biopsy) for suspicious lesions. The negative predictive value (80.0%), though relatively lower, is still clinically meaningful, as a negative result may reduce unnecessary interventions in patients with benign lesions. However, the reduced negative predictive value in the Luminal A subgroup (60%, 3/5) emphasizes that a negative FAPI-PET result should not rule out malignancy in patients with clinical or radiological suspicion for Luminal A breast cancer, particularly when lesions are small and in dense breasts. The semi-quantitative thresholds (SUVmax > 2.5 and TBR ≥ 1.5) were critical to minimizing subjective interpretation: SUVmax > 2.5 ensured detection of high-avidity malignant lesions, while TBR ≥ 1.5 addressed cases with modest SUVmax but clear contrast to normal tissue (e.g., small invasive ductal carcinoma in dense breasts). This combined approach aligns with recent FAPI-PET guidelines [12], which recommend integrating both absolute SUV and TBR to improve diagnostic accuracy. The single false-positive case (an inflammatory nodule) might be explained by FAP expression in activated fibroblasts during the inflammatory response—a known limitation of FAP-targeted imaging that has been reported in previous studies [17,18]. Notably, follow-up imaging 4 weeks after anti-inflammatory therapy confirmed complete resolution of the lesion and normalized FAPI uptake, validating the inflammatory origin and excluding malignancy. This highlights the utility of short-term follow-up imaging for distinguishing inflammatory lesions from malignant ones in cases of ambiguous FAPI uptake.

### Correlation between tracer uptake and pathological features

A key finding of our study is that by FAPI uptake parameters (SUVmax and SUVmean) did not show a significant correlation with pathological type (*P* = 0.635 for SUVmax, *P* = 0.652 for SUVmean), immunohistochemical markers (ER: *P* = 0.350 for SUVmax, *P* = 0.492 for SUVmean; PR: *P* = 0.520 for SUVmax, *P* = 0.695 for SUVmean; HER2: *P* = 0.240 for SUVmax, *P* = 0.460 for SUVmean; Ki-67: *P* = 0.270 for SUVmax, *P* = 0.375 for SUVmean), or molecular subtype (*P* = 0.587 for SUVmax, *P* = 0.603 for SUVmean). This is consistent with several recent studies: Mona CE et al. [14] reported no significant association between by FAPI uptake and ER/PR/HER2 status in breast cancer, while Katal et al. [19] found no difference in FAPI uptake among different molecular subtypes.

Notably, non-significant numerical trends and small-to-moderate effect sizes were observed across subgroups: Invasive papillary carcinoma had higher uptake than other pathological types; HER2-positive and Luminal A subtypes showed slightly higher SUVmax than Luminal B and triple-negative subtypes; ER-positive tumors had ~ 18% higher SUVmax than ER-negative tumors; and Ki-67 low-expression tumors had unexpectedly higher uptake (~21% higher SUVmax) than high-expression tumors. These trends align with potential biological mechanisms (e.g., higher stromal CAF infiltration in HER2-positive or ER-positive tumors; altered CAF activation in low-proliferation tumors) but were not statistically significant due to limited sample size and low statistical power.

Notably, critical subgroups were severely underrepresented in our cohort: the Luminal A subtype accounted for only 10.2% of breast cancer cases (n = 5), mucinous carcinoma (n = 2) and DCIS (n = 1) had extremely small sample sizes, and the HER2-low subgroup (n = 27) was underpowered relative to the overall cohort. These limitations substantially increase the risk of Type II error, as the study lacked sufficient statistical power to detect subtle differences in uptake parameters across subgroups—even if such differences exist biologically. For example, mucinous carcinoma (known for indolent biology and potentially lower stromal infiltration) may exhibit distinct uptake patterns, but the small sample size (n = 2) precluded meaningful statistical analysis. This may have introduced a Type II error and limited the generalizability of the “lack of correlation” finding. Thus, larger, multicenter studies with balanced distribution of pathological subtypes and molecular phenotypes are urgently needed to confirm whether FAPI uptake is truly independent of these features or if subtle associations exist but were not detected here.

This lack of correlation underscores the nature of FAP as a pan-tumor target: its expression is driven by CAF infiltration in the tumor microenvironment, rather than the intrinsic biological characteristics of cancer cells (e.g., hormone receptor status or proliferation index) [20,21]. Clinically, this characteristic grants FAPI-PET broad applicability across all breast cancer subtypes, including those that may be less amenable to metabolic imaging [22]—an important practical advantage for regional centers managing diverse patient populations. However, it also represents a notable limitation: unlike some molecular imaging approaches that can provide insights into tumor biology (e.g., proliferation, hormone receptor status), FAPI-PET does not convey specific biological information about the tumor itself beyond confirming malignant involvement and identifying metastases. This means it cannot replace pathological analysis or molecular profiling for guiding subtype-specific treatment decisions, such as selecting endocrine therapy or anti-HER2 agents.

Interestingly, we also observed no correlation between primary lesion size and tracer uptake (SUVmax: *r* = 0.186, *P* = 0.203; SUVmean: *r* = 0.165, *P* = 0.257), which suggests that FAP expression is not simply a function of tumor volume but reflects the extent of stromal remodeling—a key feature of malignant progression. This finding is supported by a 2023 study [23] showing that FAPI uptake correlates with the subtype of the gastric cancer characterized by high CAF content, highlighting the tracer’s ability to provide insights into the tumor microenvironment beyond anatomical measurements. However, this focus on stromal characteristics rather than tumor cell biology further reinforces that FAPI-PET’s primary value lies in detection and staging, not in delineating the biological heterogeneity of breast cancer.

### Clinical implications, comparison with recent literature, added value, and scenarios for management change

Our findings further consolidate the expanding body of evidence highlighting the clinical utility of ¹⁸F-labeled FAPI tracers in breast cancer imaging, with additional single-centre, retrospective validation from a regional tertiary care setting. Gao et al. [24] reported that [(18)F] AlF-LNC1007 exhibited superior performance relative to both [(18)F] FDG and [(18)F] AlF-NOTA-FAPI-04. This tracer demonstrated higher uptake in primary tumors, lymph nodes, and bone metastases. It also showed an enhanced tumor-to-background ratio, particularly in osseous lesions. Moreover, [(18)F] AlF-LNC1007 effectively distinguished inflammatory from metastatic lymph nodes with high specificity. Qin et al. [25] documented a case in which [(18)F]-NOTA-FAPI-04 PET/CT downstaged a 47-year-old female patient with breast cancer, thereby substantially modifying her therapeutic strategy.

Beyond confirming high diagnostic efficacy, we clarify the three core clinical roles of FAPI-PET integrated into breast cancer imaging workflows—along with its unique added value over existing modalities and specific scenarios for changing patient management: as a complementary tool for initial diagnosis of BI-RADS 4/5 lesions, it addresses suboptimal conventional imaging in dense breasts (achieving definitive diagnosis in 66.7% [8/12] of indeterminate cases) and diabetic patients (8.6% of the cohort) via blood glucose independence (avoiding fasting/glucose control delays) without serving as first-line screening; as a primary nodal staging tool, it achieves a 96.5% detection rate for metastatic lymph nodes (including internal mammary and supraclavicular nodes), modifies surgical plans in 68.8% (11/16) of patients with suspected nodal involvement, reduces staging ambiguity by distinguishing metastatic from inflammatory nodes, and helps avoid understaging and unnecessary axillary dissection; as a key niche tool for problem-solving, it addresses FDG-negative/equivocal lesions (detecting 60% of Luminal A cases), resolves BI-RADS 4 ambiguity through reliable benign/malignant differentiation (significant SUVmax difference: malignant median 13.20 vs. benign 2.13, *P* < 0.001, correctly classifying 8/9 benign lesions to reduce unnecessary biopsies), aids in distinguishing recurrent tumour from fibrosis/inflammation (pending long-term follow-up validation), and identifies unsuspected multifocal disease (8.2%, 4/49) and distant metastases (6.1%, 3/49) for staging adjustment—while its high tumour-to-background ratio in BI-RADS 3/4 dense breasts addresses the missed cancer limitations of mammography/ultrasound.

For Sichuan Provincial People’s Hospital, these findings have important practical implications. First, FAPI-PET can serve as a complementary or alternative imaging tool for patients with suspected breast cancer, especially those with dense breasts, diabetes, or low-metabolic subtypes. Second, the consistent uptake across subtypes allows for standardized imaging protocols, simplifying clinical workflow. Third, the high detection rate for lymph node metastases can help optimize surgical planning (e.g., avoiding unnecessary axillary lymph node dissection in patients with no evidence of metastasis).

### Limitations and future directions

This study has several limitations. First, as a single-center retrospective study, the sample size is relatively limited, and the pathological type distribution is skewed toward invasive ductal carcinoma (83.7%), which may limit the generalizability of results to rare subtypes. Notably, the Luminal A subtype was under-represented (n = 5), and the 40% false-negative rate in this subgroup should be interpreted cautiously due to the small sample size. Larger cohorts are needed to confirm whether this rate is representative of broader clinical practice. Second, the uneven distribution of molecular subtypes (Luminal A: 10.2%; HER2-positive: 12.2%; triple-negative: 20.4%; Luminal B: 57.1%) and small subgroup sample sizes resulted in insufficient statistical power for detecting differences in uptake parameters among subtypes. Post-hoc power analysis revealed power ranging from 29.7% to 62.3% (for detecting medium-to-large effect sizes), increasing the risk of Type II error (false negative) and limiting conclusions about the uniformity of FAPI uptake across all subtypes. This is particularly critical for rare pathological subtypes (mucinous carcinoma, DCIS) and underrepresented molecular subtypes, where even large effect sizes may have been missed due to inadequate sample size. Wide 95% confidence intervals for subgroup uptake values further confirm poor precision in these comparisons. Third, the number of benign breast lesions enrolled was extremely small (n = 9), which severely limits the statistical power to estimate specificity reliably. The wide 95% CI (51.9%–99.7%) for the reported 88.9% specificity indicates that the true specificity could range from poor to excellent, and this metric cannot be considered a robust measure of FAPI-PET’s performance in distinguishing benign from malignant lesions. This small number of benign cases also introduces potential spectrum bias, as the cohort reflects the clinical reality of a tertiary care center where BI-RADS 4/5 lesions are predominantly malignant (84.5%), rather than a community-based population with a lower pretest probability of cancer. Consequently, specificity estimates may not be generalizable to settings with a higher proportion of benign lesions (e.g., screening populations). This is a critical limitation, as accurate assessment of specificity requires a sufficiently large and diverse sample of benign lesions (e.g., fibroadenomas, adenosis, inflammatory lesions) to reflect real-world clinical scenarios. Fourth, no concurrent ¹⁸F-FDG PET/CT scans were performed in this patient cohort, so an internal head-to-head comparison between FAPI and ¹⁸F-FDG PET/CT was not feasible. This study lacked a head-to-head ¹⁸F-FDG comparator; prospective trials are needed to confirm FAPI’s superiority in low-metabolic subtypes. Thus, the relative diagnostic advantage of FAPI over the most widely used functional imaging modality remains to be confirmed. Fifth, we did not include long-term follow-up data, so the value of by FAPI in treatment response monitoring and prognosis prediction cannot be addressed here. Sixth, individualized dose–exposure relationships (e.g., the correlation between administered tracer activity, patient demographics, and radiation dose) were not analyzed, and this aspect should be explored in future work to address radiation protection concerns and optimize clinical dosing strategies. Seventh, a key limitation of FAPI-PET identified in this study is its inability to distinguish between breast cancer subtypes or reflect intrinsic tumor biological features (e.g., hormone receptor status, proliferation index). This limits its utility for providing biologically relevant information to guide personalized treatment decisions, which requires integration with pathological analysis and other molecular profiling tools. Eighth, while we identified potential management-changing scenarios, prospective validation is needed to quantify the impact of FAPI-PET on treatment decisions, healthcare costs, and patient outcomes (e.g., recurrence-free survival).

Future research should address these limitations by: (1) Conducting prospective, multicenter studies to validate these findings with a more balanced distribution of pathological subtypes, particularly increasing the representation of Luminal A cases and expanding the enrollment of benign breast lesions to ensure robust estimation of specificity; (2) Designing comparative trials to explore the diagnostic performance of FAPI-PET relative to other imaging modalities (e.g., ¹⁸F-FDG PET/CT, MRI) in small lesions (<1 cm) and dense breast tissue, with a focus on Luminal A and other low-metabolic subtypes; (3) Exploring optimized imaging protocols for FAPI-PET in high-risk subgroups, such as adjusting tracer dose, acquisition time, or reconstruction algorithms to enhance detection of small, low-avidity lesions; (4) Investigating the utility of quantitative uptake parameters (e.g., SUVmax change) for monitoring neoadjuvant chemotherapy response; (5) Investigating the correlation between FAPI uptake and patient outcomes (e.g., recurrence-free survival) to establish its prognostic value; (6) Exploring combinations of FAPI-PET with other molecular imaging tracers (e.g., hormone receptor-targeted tracers) or liquid biopsies to enhance the ability to simultaneously detect lesions and characterize tumor biology, thereby improving the integration of imaging into personalized treatment workflows; and (7) Conducting prospective management impact studies to confirm that FAPI-PEt alters treatment decisions in the scenarios identified and improves clinical outcomes.

## Conclusions

FAPI-PET exhibits high sensitivity and accuracy in breast cancer diagnosis, with effective detection of lymph node metastases. In this single-center retrospective cohort, its uptake did not show a significant correlation with breast cancer pathological features or molecular subtypes, and it is not affected by blood glucose levels. However, these findings are limited by the small sample size of rare pathological subtypes (e.g., mucinous carcinoma, DCIS) and underrepresented molecular subtypes (e.g., Luminal A), which precludes definitive conclusions about “uptake independence”. Its core clinical roles—complementary initial diagnosis in high-risk subgroups, primary nodal staging, and problem-solving in equivocal cases—address key unmet needs in existing imaging workflows. These characteristics make it a versatile and reliable imaging tool for breast cancer diagnosis and staging in regional tertiary care settings based on single-centre, retrospective evidence, but further validation in larger, balanced cohorts is needed to confirm the universality of its uptake patterns across all breast cancer subtypes.

## Supporting information

S1 Table Baseline characteristics of the study cohort. (DOCX)
