# Supplementary material for: Al18F-NOTA-FAPI-04 PET/CT for diagnosis of breast cancer and its correlation with pathological features: A single-center retrospective study
Source: PLoS One. 2026 Feb 12;21(2):e0342769. doi: 10.1371/journal.pone.0342769 (PMC12900296; doi:10.1371/journal.pone.0342769)
Supplement: S1 Table — (DOCX) [file pone.0342769.s001.docx]

**S1 Table. Baseline characteristics of the study cohort.**

| **Characteristic** | | **Total Cohort (n=58)** | **Breast Cancer (n=49)** | **Benign Lesions (n=9)** |
| --- | --- | --- | --- | --- |
| **Age, median (IQR), years** | 54 (47–62) | | 55 (48–63) | 51 (44–58) |
| **Menopausal status, n (%)** |  | |  |  |
| **- Premenopausal** | 21 (36.2) | | 17 (34.7) | 4 (44.4) |
| **- Postmenopausal** | 37 (63.8) | | 32 (65.3) | 5 (55.6) |
| **BMI, median (IQR), kg/m²** | 23.8 (21.5–26.3) | | 24.0 (21.8–26.5) | 23.2 (20.9–25.7) |
| **Diabetes mellitus, n (%)** | 5 (8.6) | | 4 (8.2) | 1 (11.1) |
| **Pathological diagnosis, n (%)** |  | |  |  |
| **- Breast cancer** | 49 (84.5) | | 49 (100) | - |
| **- Benign lesions** | 9 (15.5) | | - | 9 (100) |
| **- Fibroadenoma** | - | | - | 4 (44.4) |
| **- Breast hyperplasia** | - | | - | 3 (33.3) |
| **- Inflammatory nodule** | - | | - | 2 (22.2) |

Note: BMI = body mass index; IQR = interquartile range.
